# Advances and Application of Polyphenol Oxidase Immobilization Technology in Plants

**DOI:** 10.3390/plants14152335

**Published:** 2025-07-28

**Authors:** Fang Zhou, Haiyan Lin, Yong Luo, Changwei Liu

**Affiliations:** 1 School of Chemistry and Environmental Sciences, Xiangnan University, Chenzhou 423000, China; fangzhou@xnu.edu.cn; 2 Key Laboratory of Tea Science of Ministry of Education, Hunan Agricultural University, Changsha 410128, China; linhy668@163.com; 3 School of Life and Health Sciences, Hunan University of Science and Technology, Xiangtan 411201, China

**Keywords:** plant polyphenol oxidase, immobilization technology, immobilization material, application

## Abstract

Polyphenol oxidase (PPO) is a metalloproteinase widely present in plant organelles that plays crucial roles in photosynthesis, pest and disease resistance, growth and development, and flower color formation. Due to the high cost and reuse difficulties of plant PPO in applications, immobilization has emerged as a key technology to improve its stability, recyclability, and reusability. Immobilized plant PPO has been widely used in environmental and detection fields. This review examines different immobilization methods and carrier materials for plant PPO and summarizes its applications in wastewater treatment, biosensor detection, food preservation, and theaflavin synthesis. Finally, current challenges and future opportunities for immobilized plant PPO are discussed.

## 1. Introduction

Polyphenol oxidase (PPO) is a widely distributed metalloproteinase found in plants, animals, and microorganisms [1,2]. Its catalytic active site primarily consists of Cu atoms, which catalyze the formation of quinones from polyphenols under aerobic conditions. These quinones eventually aggregate to form water-insoluble polymer precipitates that are removed [3,4]. In plants, multiple PPO-expressing genes have significant roles in photosynthesis, pest and disease resistance, growth and development, and flower color formation [5,6,7]. Mushrooms, as PPO-rich fungal substances, are often classified with plants by researchers due to their specificity [8]. With the decoding of PPO gene sequence and in vitro expression of genes from sources like banana, apple, potato, mushroom, and tea, plant PPO proteins can be easily prepared and have a wide range of food, environmental, and detection applications [9,10,11]. However, the high cost and reuse difficulties of free PPO hinder its industrial applications, as it cannot be recycled and is inactivated at extreme pH and high temperatures [12,13]. This has accelerated research into methods for improving PPO stability and recycling.

Enzyme immobilization techniques have been developed as biocatalysis demands catalytic performance and stability from enzymes. This technology anchors enzymes to solid supports via physical or chemical interactions, overcoming the inherent limitations of natural enzymes in industrial applications [14,15]. Its core advantages are reflected in three aspects. Firstly, the microenvironmental barrier constructed by immobilization effectively delays the destruction of the enzyme conformation by environmental factors such as pH, temperature, and solvent, avoids inactivation of the enzyme, and improves the stability of the enzyme. For example, Al-Harbi et al. (2023) covalently immobilized β-galactosidase at an optimal temperature of 60 °C, which is 10 °C higher than that of the free enzyme [16]. Secondly, the utilization of responsive carrier or reversible carrier adsorption makes the reproducible immobilization of enzyme or carrier possible. For example, Bao et al. (2021) reversibly adsorbed and desorbed the enzyme through non-covalent interactions, enabling the reuse of the enzyme and materials [17]. Finally, the enzyme immobilized in a carrier can be recycled multiple times, which is not possible with free enzymes. For example, Ulu et al. (2020) covalently immobilized laccase, which retained 50% of its initial relative activity after 20 cycles [18]. These enhanced properties—improved stability, reusability, and recyclability—drive the adoption of immobilized enzymes in biological, food, and environmental remediation applications. Consequently, efficient enzyme immobilization systems present a promising strategy for boosting enzyme stability and operational recyclability.

Enzyme immobilization techniques have enhanced the stability, recoverability, and reusability of PPO [19,20]. Immobilizing PPO on a carrier improves its stability, converting it into a non-homogeneous phase catalyst that can be recycled and reused after catalyzing reactions in a bioreactor. For example, Zeng et al. (2019) immobilized apple-extracted PPO on mesoporous silica, which improved enzyme stability and retained 40% of the original activity after 10 consecutive cycles [21]. Immobilization has enabled a wide range of PPO applications in industries such as environmental treatment, biosensors, and theaflavin synthesis [5,9,22,23]. A good immobilization system reduces enzyme inactivation, increases enzyme adsorption, improves stability, and enhances reactor reusability [13,19]. Therefore, selecting appropriate immobilization methods and support materials, along with optimizing immobilization parameters, is essential for the successful application of immobilized PPO [24,25,26]. Collectively, previous research on immobilized PPO provides valuable insights that can inspire further studies to advance immobilized PPO technology and applications.

To provide a comprehensive discourse for subsequent research on immobilized plant PPO, this review presents an overview of the latest advances in immobilization methods and carrier materials for plant PPO (including mushrooms). The applications of immobilized plant PPO in food, environmental, and testing fields are then discussed. Finally, current challenges and future opportunities associated with immobilized plant PPO are examined.

## 2. PPO in Plants

### 2.1. Sources and Functions of PPO in Plants

Plants, as an energy storage substance, are one of the major sources of PPO. In plants, most PPOs are present in locations such as chloroplasts of photosynthetic cells and non-photosynthetic organelles, bound to membranes [2]. Native PPO exists in an inactive state; but after cutting or damage, PPO meets with oxygen and combines with phenolics to undergo a browning reaction [27]. There are differences in PPO depending on the source, variety, and maturity of the raw material. For example, in tea trees, PPO has two forms of existence. The former is predominantly found in chloroplasts, bound to the endocyst membrane system in an insoluble state, and distributed in a latent inactive form [28,29]. The latter is found mainly in non-photosynthetic tissue vesicles in a soluble state [30]. As the tea tree matures and ages, the soluble PPO is gradually converted to insoluble membrane-bound PPO, and then the PPO activity spontaneously decreases [31]. In fresh apples, on the other hand, PPOs are almost exclusively present in the membranes of organelles such as chloroplasts and mitochondria, or in soluble fractions, and exhibit different enzymatic properties [32,33]. For example, Xu et al. (2011) sequenced the genome sequence of potato and isolated nine genes for PPOs: StPOTP1, StPOTP2, StPOT32, StPOT33, StPOT72, and StuPPO5-StuPPO9 [34]. Based on the magnitude of the effect of PPO activity, Bøjer Rasmussen et al. (2021) characterized the two main PPOs responsible for oxidase activity in potato tuber tissues and showed that both PPOs had an optimal pH of 5, were stable in the pH 6 to 11 range, and had an optimal temperature of 40 °C [35]. Similarly, a variety of PPO gene sequences were present in bananas, apples, and tomatoes, which have optimal enzyme activity for PPO at pH values ranging from 5 to 8 and at temperatures ranging from about 20 °C to 50 °C. Specifically, PPO is widely available and abundant in plants. Mushrooms are abundant and readily available as part of the diet consumed by humans. When mushrooms are cut, their cut flesh turns black when exposed to air, mainly due to the browning reaction of PPO. *Agaricus bisporus* is the most common commercially available species, and its optimum temperature and pH are similar to those of plants due to the fact that it is located in essentially the same environment as plants [8,36,37].

Plant PPO typically exists in both soluble and membrane-bound forms, exhibiting distinct enzymatic properties that reflect their functional diversity within plant cells [38]. PPO participates in plant secondary metabolic processes and indirectly synthesizes plant secondary metabolites, which have a variety of biological functions such as acting as antioxidants and influencing plant development [3,39]. For example, PPO catalyzes the production of theaflavins from catechins that affect the quality and flavor of tea [5]. Furthermore, PPO is also involved in defense and disease resistance metabolic pathways in plants [40,41]. When the plant body is subjected to mechanical damage or pathogen infestation, the separated PPO comes into contact with phenolic substrates and catalyzes the production of quinones. On the one hand, quinones have antimicrobial properties and can inhibit the growth and reproduction of pathogenic microorganisms [42]; on the other hand, it can also form melanin to enhance the strength and rigidity of the cell wall, forming a physical barrier that prevents invasion of pathogens [43]. Notably, in fruit and vegetable storage, PPO catalyzes the oxidation of phenolics to produce quinones and polymers, generating tissue browning, leading to darkening of plant tissues, and affecting appearance quality and market value [44]. Collectively, PPO functions as an integral regulator of plant growth, stress responses, and quality attributes through its involvement in multiple metabolic pathways.

### 2.2. Classification of PPO in Plants

**Figure 1:**
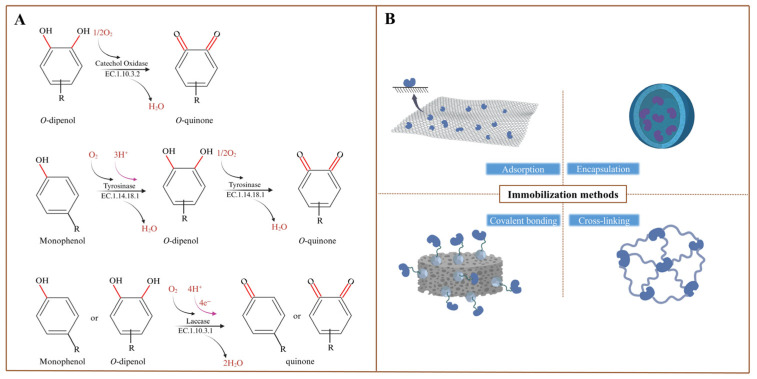
(**A**) Catalytic activities of catechol oxidase, tyrosinase, and laccase; (**B**) methods of immobilization of plant PPO.

PPOs, as oxidoreductase enzymes from a wide range of sources, are divided into three main groups based on their catalytic activity and substrate specificity: tyrosinases (EC 1.14.18.1), catechol oxidases (EC 1.10.3.1), and laccases (EC 1.10.3.2) (Figure 1A) [29,45]. Tyrosinase and catechol oxidase are binuclear type III Cu ion-centered enzymes with an active site for two Cu ions (Cu_A_ and Cu_B_), both copper atoms coordinated by three histidine residues [46]. Although the two enzymes have the same active site and are structurally similar, the activity of catalyzing the substrate is markedly different [45,47]. Researchers have different understandings and speculations about the difference in the mechanism, but some of the catalytic activity and the mode of conversion of the substrate are unified in both enzymes. Tyrosinase is a bifunctional catalytically active enzyme with monophenolase and diphenolase catalytic activities. Its monophenolase activity catalyzes the o-hydroxylation of monophenolic compounds, followed by diphenolase activity that oxidizes o-diphenolic compounds to the corresponding o-quinones and water [48,49]. On the other hand, catechol oxidase, a widely distributed PPO in plants with only diphenolase activity, catalyzes the oxidation of o-diphenols to the corresponding o-quinones and water [50]. The catalytic Cu center of laccase, a typical representative of type III copper proteins, typically contains four Cu ions. The four Cu ions constitute the catalytic core of the intermolecular electron transfer reaction of laccase, which consists of one type I Cu ion (T1-Cu), one type II Cu ion (T2-Cu), and two type III Cu ions (T3-Cu: T3-Cu_a_, T3-Cu_b_), respectively. Among them, T2-Cu is closer to T3-Cua and T3-Cub, forming a trinuclear cluster responsible for catalyzing the reduction of oxygen to obtain water, and is about 10 Å or more away from T1-Cu, which is responsible for obtaining electrons [51]. T1-Cu is responsible for obtaining single electrons from the substrate and then transferring them to the trinuclear cluster where substrate oxidation occurs. T2-Cu is a one-electron acceptor and T3-Cu is a two-electron acceptor where oxygen is reduced to water [52,53]. In plants, PPOs contribute critically to pigment biosynthesis and cell wall formation [54]. In addition, based on the enzyme activity and substrate specificity of plant and mushroom PPOs, they have been used by many researchers in the fields of food, medicine, and detection [21,55].

## 3. Plant PPO Immobilization

In the application of PPO, there are drawbacks such as poor stability, activity, and reusability [13]. To overcome these limitations, immobilization techniques have been used. With advances in nanotechnology and material science, more nanomaterials are being used as carriers for PPO immobilization [56,57]. The carrier’s nature directly relates to PPO properties, such as activity, stability, and reusability [58]. Additionally, the choice of immobilization technique determines how PPO interacts with the carrier, including physical methods (adsorption and embedding) and chemical methods (covalent binding and cross-linking) (Figure 1B) [13]. The catalytic performance of immobilized PPO varies with different immobilization strategies, and the choice of technique depends on the nature of PPO and substrate orientation [5]. And after PPO immobilization, often a decrease in enzyme activity occurs, and the rate at which the initial PPO activity can be retained is inextricably linked to the choice of material and the immobilization method used. PPO immobilization methods and applications derived from fungal and bacterial gene expression have been reported and reviewed [13,20,59,60,61,62]. In contrast, plant PPO is a rich source and has been extensively immobilized and studied, but it has not been reviewed and gaps exist. Therefore, it is necessary to summarize plant PPO immobilization techniques to select suitable methods for subsequent studies. Hereafter, polyphenol oxidase is referred to as PPO.

### 3.1. Physical Methods

Physical adsorption is a relatively simple method of immobilization and is easier to operate. Under certain conditions, PPO will be adsorbed on the surface or in the pores of the carrier through physical forces (van der Waals forces, classical interactions, etc.), which is a simple operation with higher retention of enzyme activity compared to encapsulation [63]. Physical encapsulation, which involves embedding PPO in a carrier matrix such as a gel or polymer, results in the enzyme being physically trapped while the substrate remains free to diffuse. This method is highly resistant to interference compared to adsorption [13]. This also gives physical methods obvious advantages in operation and cost, which is more favorable for large-scale applications compared with some immobilization means that require the introduction of special chemical reagents or complex cross-linking reactions. Moreover, the use of physical adsorption and physical embedding to immobilize PPO can maintain the natural structure of the enzyme to a large extent, so that PPO can still maintain efficient catalytic activity after immobilization, which is crucial for biocatalysis, food processing, and other fields [20,64]. However, both physical immobilization methods also have significant drawbacks. The enzyme–carrier interaction is relatively weak and less specific [14,65]. On the one hand, since the enzyme molecules are mainly adsorbed on the carrier by physical forces, it is easy to make the enzyme molecules detach from the carrier when disturbed by external physical and chemical factors, which leads to the decrease in the stability of the immobilized enzyme. On the other hand, poor specificity means that the binding of the enzyme molecule to the carrier is random, and it is impossible to avoid the masking of the active center of the enzyme as well as the shedding of the enzyme. Table 1 summarizes current advances in research on physical methods for immobilizing PPO.

#### 3.1.1. Adsorption

Physical adsorption is the simple attachment of an enzyme to an insoluble carrier’s outer surface based on weak interactions (including ionic interactions, van der Waals forces, or dipole interactions) on the carrier surface [63,66]. It has wide applicability and circumvents carrier regeneration problems by removing the inactivated enzyme and introducing a new enzyme. Therefore, physical adsorption is the first choice for enzyme immobilization. However, it can be affected by the external liquid environment, such as pH and ionic strength, leading to enzyme damage and inactivation [13].

Mesoporous silica serves as an exemplary support due to its high surface area, substantial pore volume, and tunable pore dimensions that optimize enzyme adsorption [67,68]. Iriarte-Mesa et al. (2023) immobilized *Agaricus bisporus* PPO (Ab PPO) into SBA-15 mesoporous silica by liquid adsorption and compared it with immobilizing Ab PPO into aminocarbonated SBA-15 silica mesopores by electrostatic interactions (negatively charged Ab PPO and positively charged aminocarbonated SBA-15 silica) [69]. Both methods improved pH stability, thermal stability, and regeneration, achieving simple PPO preparation, low production cost, and reusable carriers. However, to achieve the same immobilization effect, the aminated SBA-15 type has lower enzyme adsorption than the SBA-15 type. Reducing the enzyme carrier’s size generally preserves enzyme flexibility and enhances loading, leading to increased immobilized enzyme activity [70]. Chitosan/montmorillonite (CTS/MMT) nanocomposites, commonly used as adsorbents, form intercalated structures with a high specific surface area, good physicochemical stability, and numerous adsorption sites [71,72], arousing great interest in their use as enzyme carriers. [72] immobilized PPO on CTS/MMT (IPPO) material and CTS-/MMT (IPPO-Au) composites containing Au nanoparticles (AuNPs), effectively improving PPO catalytic activity [72]. Compared with IPPO, IPPO-Au enzyme activity increased by 13.4 × 10^3^ U/g with higher thermal stability and reproducibility. IPPO-Au was synthesized based on the reaction of HAuCl_4_ and CTS on CTS/MMT composites with AuNPs, incorporating AuNPs’ high affinity and biocompatibility. The smaller size of AuNPs maintains enzyme flexibility and improves activity. The team then optimized PPO immobilization parameters. Compared with pre-optimization of IPPO-Au, the immobilization efficiency increased by 44.42% and enzyme activity increased by 1.09 × 10^3^ U/mg after optimization [72]. Subsequently, the removal of phenolic compounds from wastewater pollutants was investigated [71]. Graphene paper has been used in composites with immobilized enzymes for sensing detection due to its good chemical stability and mechanical flexibility. Kıranşan et al. (2020) vacuum-filtered graphene oxide (GO) to obtain GO paper, which was used with deposited polyglycine to form a poly(glycine)/reduced GO (rGO) composite [11]. The material then adsorbed PPO to form a PPO-poly(glycine)/rGO sensor, which maintained 80% electrocatalytic activity with high stability and reproducibility after 30 cycles. It was used to detect catechols in water samples with a detection limit as low as 0.07 μmol/L.

Immobilization of PPO is often associated with increased enzyme activity and stability for applications such as removal of phenolic compounds and assays [22,68]. However, some researchers focus on inhibiting PPO activity, as PPO release causes enzymatic browning that affects the flavor, color, and nutritional value of fruits [27]. Inhibiting PPO activity through nanocomposites is crucial for the fruit and vegetable industry. Inhibiting enzymatic browning often requires adsorbing or isolating PPO from related phenolics in fruits and vegetables. Good biocompatibility and strong adsorption are important for immobilized materials. Corell Escuin et al. (2017) adsorbed various mesoporous silica materials (SBA-15, SBA-3, and MCM-48) with PPO via electrostatic interactions and found that SBA-15 could adsorb 50% of PPO within 15 min, inhibiting 50% of PPO activity [25]. The electrically charged nature and large pore size of SBA-15-type mesoporous silica at pH leads to higher and faster loading capacity, making it more suitable for inhibiting PPO in fresh fruits and fruit juices. In addition to stability, GO is biocompatible and irreversibly binds to enzymes through electrostatic interactions [73], inhibiting PPO activity. Gür et al. (2019) immobilized PPO on GO and rGO to form GO-PPO and rGO-PPO with good storage and thermal stability that can be removed from fruits and vegetables by adsorption during PPO activity [74].

#### 3.1.2. Encapsulation

Physical encapsulation captures and immobilizes the enzyme in a three-dimensional carrier material without chemical bonding or structural changes, effectively avoiding enzyme overflow, inactivation, and mixing of reaction products. Sodium alginate (SA), a natural polymer exhibiting inherent hydrophilicity, biodegradability, and biocompatibility [75], serves as an effective immobilization matrix. Its capacity to form hydrogels through cross-linking with multivalent cations or bifunctional agents enables efficient enzyme encapsulation [76]. Edalli et al. (2016) embedded PPO within SA, SA-polyvinyl alcohol (PVP), and SA-polyvinyl alcohol-silver nanoparticles (SA-PVA-AgNPs) [77]. Compared to free PPO, immobilized PPO showed significantly improved thermal stability, retaining 72% activity at 50 °C versus just 26% for free PPO. SA-PVA-AgNPs-PPO was reused more than 12 times with unchanged phenolic degradation activity. Considering SA’s poor mechanical stability in excess acid or base, which may lead to low immobilization efficiency and embedding leakage, Almulaiky et al. (2022) mixed SA with zinc oxide nanoparticles (ZnO NPs) to obtain SA-ZnO NPs, and embedded PPO inside [78]. Compared to free PPO, immobilized PPO showed 782 U/mg higher activity, a 10 °C increase in optimal temperature, enhanced substrate affinity, and 69% activity retention after 10 cycles [78]. The enhanced properties may be attributed to the strong interaction between SA’s carboxyl group and ZnO. Embedded PPO has also been researched for phenolic compound detection. Based on PPO’s enzymatic reaction with phenolic compounds, Mishra et al. (2020) proposed a fiber optic sensor for detecting catechols in aqueous solution using PPO encapsulated in polyacrylamide gel on the optical fiber [79]. The sensor showed good reproducibility and stability with a maximum sensitivity of 0.0088 nm/µM for catechol sensing. Micheloni et al. (2018) homogeneously immobilized PPO in thin layer chromatography by gel-embedding and measured the reaction rate of PPO with phenolic substrates in response to inhibitor addition to assess PPO activity inhibition [80]. This self-expressive system provides a rapid assay for finding optimal inhibitors of enzymatic browning produced by different PPO sources.

**Table 1:** Summary of physical methods for immobilized plant PPO.

Physical Methods	PPO Source	Materials	Payload Capacity	Retention Rate of Enzyme Activity	pH Stability	Temperature Stability	Storage Stability	Cyclic Stability	Reference
Adsorption	Mushroom	Transition metal carbides/Yttrium oxide	-	100%	-	-	Retained 84.1% of initial activity after 2 weeks storage at 4 °C	After 10 cycles of measurement, the RSD was 2.34%	[81]
	Mushroom	SBA-15 silica	7.20 μmol·g^−1^ silica	91%	At pH = 3, the free enzyme was inactivated and the immobilized enzyme maintained 25% of the initial enzyme activity	At 70 °C, the free enzyme was inactivated and the immobilized enzyme maintained 22% of the initial enzyme activity	Retained 66% of initial activity after 6 months storage at 4 °C	After 15 cycles, 63% of initial enzyme activity was retained	[69]
	Kombucha tea	Carbon paste/Mineral oil	-	-	-	-	-	-	[82]
	Solanum lycocarpum	Carbon paste/Mineral oil	-	-	-	-	Retained 68.4% of initial activity after 15 days storage at 4 °C	After 5 cycles, 58.7% of the initial enzyme activity was retained	[83]
	Mushroom	Chitosan/Organic rectorite	17.30 mg/g	-	-	-	-	After 10 cycles, 60.3% of the initial enzyme activity was retained	[22]
	Mushroom	Chitosan/Montmorillonite	13.17 mg/g	-	At pH 7, free PPO and immobilized enzyme retained 88.4% and 96.6% of their initial activity at 8 h, respectively.	E_d_ is 56.77 kJ/mol and 65.57 kJ/mol for free and immobilized enzymes, respectively (E_d_: energy required for enzyme inactivation)	-	After 10 cycles, 52.1% of the initial enzyme activity was retained	[72]
		Chitosan gold nanoparticles/Montmorillonite	18.93 mg/g	-	At pH 7, free PPO and immobilized enzyme retained 88.4% and 98.4% of their initial activity at 8 h, respectively	E_d_ is 56.77 kJ/mol and 72.24 kJ/mol for free and immobilized enzymes, respectively	-	After 10 cycles, 59.1% of the initial enzyme activity was retained	
	Potato	Poly(glycine)/Reduced graphene oxide	24 µg/cm^2^	12%	-	-	Retained 85% of initial activity after a week storage at 4 °C	After 10 cycles, optimal catalytic activity is maintained	[11]
	Ipomoea batatas	Strontium copper oxide/Polypyrrole nanotubes	-	-	-	-	Retained 81% of initial activity after 18 days storage at 4 °C	After 6 cycles of measurement, the RSD was 0.76%	[84]
	Potato	Filter paper	-	-	-	-	Retained 98.7% of initial activity after 4 weeks storage at 4 °C	After 3 cycles of measurement, the RSD was 1.6%	[85]
	Grape	Graphene oxide	--	-	-	-	-	-	[74]
	Banana	Graphite powder/Paraffin	-	-	-	-	Retained 75% of initial activity after 40 days storage at 4 °C	After 6 cycles of measurement, the RSD was 3.2%	[86]
	Sapota	Graphite/Polypyrrole/Silver nanoparticles	-	-	-	The optimum temperature is increased to 40 °C	Retained 82% of initial activity after 2 weeks storage at 4 °C	-	[87]
	Jenipapo	Carbon paste/Mineral oil	-	-	-	-	Retained 88.22% of initial activity after 15 days storage at 4 °C	-	[88]
	Jurubeba	Carbon paste/Mineral oil	-	-	-	-	Retained 87.79% of initial activity after 6 weeks storage at 4 °C	-	[89]
	Sapota	Graphite/Graphene nanoribbons/Silver nanoparticles					Retained 80.82% of initial activity after 25 days storage at 4 °C	After 15 cycles of measurement, the RSD was 3.2%	[90]
	Sapota	Graphite/Graphene nanoribbons/Silver nanoparticles	-	-	-	-	Retained 90% of initial activity after 6 days storage at 4 °C	-	[91]
	Mushroom	SBA-15 silica, SBA-3 silica, MCM-48 silica	300 mg/g, 100 mg/g, 140 mg/g	-	-	-	-	-	[25]
	Banana	Graphite powder/Hydrogel	-	-	-	-	--	-	[92]
	Pear	Tyrosinase					Retained 70% of initial activity after 3 weeks storage at 4 °C	After 21 cycles, 70% of the initial enzyme activity was retained	[93]
	Potato	Diethylaminoethyl/Cellulose fibers	-	-	pH activity distribution, the immobilized enzyme has a wider distribution than the free enzyme	At 0–90 °C, the free enzyme is more sensitive than the immobilized enzyme	-	After 21 cycles, 55% to 60% of the initial enzyme activity was retained	[94]
Entrapment	Mushroom	Polyacrylamide gel	-	-	-	-	Retained 100% of initial activity after 4 weeks storage at 4 °C	-	[79]
	Apple	Thin layer chromatography	-	-	-	-	-	-	[80]
	Mushroom	Sodium alginate/Polyvinyl alcohol	-	-	At pH 6–10, the free enzyme is more sensitive to pH than the immobilized enzyme	At 30–50 °C, the free enzyme is more sensitive than the immobilized enzyme	-	After 8 cycles, 100% of the initial enzyme activity was retained	[77]
	Mushroom	Sodium alginate/Polyvinyl alcohol/Silver nanoparticles	-	-	At pH 6–10, the free enzyme is more sensitive to pH than the immobilized enzyme	At 30–50 °C, the free enzyme is more sensitive than the immobilized enzyme	-	After 12 cycles, 100% of the initial enzyme activity was retained	[77]
	Quince	Calcium alginate	-	-	pH activity distribution, the immobilized enzyme has a wider distribution than the free enzyme	The optimum temperature for free enzyme is 30 °C, and in the case of immobilized enzyme, it is 35 °C	-	-	[95]

Relative standard deviation: RSD.

### 3.2. Chemical Methods

The main chemical methods for immobilizing PPO are chemical cross-linking and covalent bonding. The covalent-bonding method utilizes the reactive groups on the carrier to react with the amino group, carboxyl group, and other functional groups in the PPO, and connects them through covalent bonds [96]. The chemical cross-linking method uses a cross-linking agent, such as glutaraldehyde, to cross-link PPO with the carrier, or between PPOs [5]. Both strategies establish stable covalent linkages between the enzyme and its support. This tight binding confers high stability to the immobilized enzyme, minimizes enzyme leaching during operation, and extends its operational lifetime. However, chemical modification has some shortcomings [5,97]. On the one hand, chemical modification requires the use of specific chemical reagents and complex operation procedures, which increases the cost of immobilization. On the other hand, chemical modification may change the spatial structure of PPO and decrease the enzyme activity. In addition, the conditions of chemical modification are difficult to control, and different combinations of PPO and carriers may require optimization of the reaction conditions. Current advances in chemical methods for immobilizing PPO are summarized in Table 2.

#### 3.2.1. Cross-Linking

The cross-linking method has provided good stability in various applications of immobilized enzymes [13,98]. This approach employs bifunctional agents (e.g., glutaraldehyde or glyoxal) that form covalent bonds with amino acid residues in PPO, significantly enhancing enzyme stability [99]. Currently, cross-linked immobilized PPO has been used for the removal or detection of phenolic compounds and has shown good characteristics. Li et al. (2017) introduced glutaraldehyde (GA), a commonly used protein cross-linking agent, to the surface of carbon nanoelectrodes and then immobilized PPO by cross-linking [100]. The immobilized PPO maintained 88% of its original activity after 30 days, demonstrating excellent stability. It also showed good substrate affinity and reproducibility in the phenolic substrate reaction, enabling rapid detection of phenolic compounds. Magnetic nanoparticles, characterized by a large surface area ratio, high enzyme loading, and easy recovery in the presence of a magnetic field, are commonly used for enzyme immobilization [101,102]. Marjani et al. (2021) encapsulated magnetic nanoparticles with alginate to prevent their oxidation, then added GA to bind to the material’s surface and cross-link with PPO [10]. The immobilized PPO showed a 34% increase in enzyme activity after 12 days of storage and better pH stability compared to free PPO. After seven cycles of removing phenolic compounds, the immobilized PPO retained 41% of its original activity, exhibiting good reproducibility and recovery stability. As a key enzyme catalyzing theaflavin synthesis in tea trees [103], PPO immobilization is necessary to enhance its utilization efficiency. Lei et al. (2017) encapsulated magnetic nanoparticles with chitosan, and GA covalently bound to the material’s surface was used to immobilize purified PPO from pears [104]. The immobilized PPO loading on the magnetic nanoparticles was as high as 145 μg/mg, and the immobilized PPO-synthesized theaflavin retained 85% of its initial activity after eight consecutive cycles. This is similar to the results of Zeng et al. (2019) who cross-linked purified apple PPO on mesoporous silica via GA, showing good activity, stability, and reusability [21].

#### 3.2.2. Covalent Binding

The covalent-binding method relies on the covalent attachment of chemically functional groups (e.g., amino, hydroxyl, sulfhydryl, and imidazole groups) of the support carrier to the nucleophilic groups of the enzyme (α-carboxylic group at the C-terminus, α-amino group at the N-terminus, indole ring, etc.), preventing the enzyme from being readily dislodged [105]. However, this method requires complex steps, long reaction times, and can significantly limit structural changes in the enzyme [106]. Carriers such as chitosan, cellulose, and porous materials are initially surface-modified using cross-linking reagents like GA or metal nanoparticles. Zhong et al. (2021) prepared PPO immobilization-supported chitosan/organic rectorite (CTS/OREC) composites by embedding CTS in OREC [22]. Immobilization with PPO was carried out through two strategies: direct adsorption (APPO) and covalent binding (CPPO) via GA activation of the CTS/OREC composites. The effect of the immobilization strategies on PPO’s catalytic activity for removing phenolic compounds was explored. CPPO’s enzyme activity was reduced by 7.45 × 10^3^ U/g and enzyme sequestration increased by 9.2 mg/g compared to APPO, demonstrating the typical advantages and disadvantages of physical and chemical immobilization strategies.

### 3.3. Mixed Methods

Given the inherent limitations of individual immobilization strategies, integrating multiple approaches presents a promising strategy to enhance PPO’s operational stability, catalytic activity, reproducibility, and application scope. For example, enzymes can be pre-immobilized on composite carrier surfaces by physical adsorption or embedding, then form strong interactions with the enzymes through cross-linking or covalent binding [107]. Zheng et al. (2019) constructed polyaniline–porous polyacrylonitrile–graphene nanomaterials with good biocompatibility, high stability, and strong adsorption capacity [108]. They first utilized porous polyacrylonitrile’s adsorption capacity to encapsulate PPO into the nanomaterials, then cross-linked GA with PPO. This enabled rapid detection and sensing of polyphenols by PPO with excellent sensitivity and selectivity. Mondal et al. (2017) immobilized PPO on reduced graphene oxide/chitosan (rGO/CTS) composite nanomaterials containing gold nanoparticles (AuNPs) by CTS adsorption and GA cross-linking [109]. The immobilized PPO showed enhanced affinity for phenolic substrates compared to free PPO. In conclusion, future research must seek a combination of high-performance carriers and diverse immobilization methods to obtain immobilized PPOs with high stability, enzymatic activity, and reproducibility for applications such as detecting and removing phenolic pollutants, bleaching dyes, and environmental remediation.

**Table 2:** Summary of chemical methods for immobilized plant PPO.

Physical Methods	PPO Source	Materials	Payload Capacity	Retention Rate of Enzyme Activity	pH Stability	Temperature Stability	Storage Stability	Cyclic Stability	Reference
Cross-linking	Tea tree	Polyethylene glycol	-	-	-	-	-	After 3 cycles, 80% of the initial enzyme activity was retained	[103]
	Mushroom	3,4-ethylenedioxythiophene/Graphene oxide	-	-	-	The optimum temperature is increased to 45 °C	Retained 74% of initial activity after 25 days storage at 4 °C	After 30 cycles, 50% of the initial enzyme activity was retained	[23]
	Green tea	Alginate/Magnetic Fe_3_O_4_/Glutaraldehyde	-	-	At pH 4–11, the free enzyme is more sensitive to pH than the immobilized enzyme	At 40–80 °C, the free enzyme is more sensitive than the immobilized enzyme	Retained 87% of initial activity after 12 days storage at 4 °C	After 7 cycles, 41% of the initial enzyme activity was retained	[10]
	Mushroom	Porous graphene/Polypyrrole					Retained 65% of initial activity after 60 days storage at 4 °C	After 21 cycles, 80% of the initial enzyme activity was retained	[110]
	Mushroom	NH_2_-SBA-15 silica/L-tyrosine/Gold nano-particles/Glass carbon	-	-	-	-	Retained 92.6% of initial activity after 12 days storage at 4 °C	-	[111]
	Mushroom	Thiophene-3-boronic acid/Gold nanopartilces/Glutaraldehyde	-	-	-	-	-	-	[112]
	Ipomoea batatas	Babassu mesocarp nanoparticles/Glutaraldehyde	-	-	-	-	-	After 30 days of cycling, the total current loss was 7.5%	[113]
	Mushroom	Pt/CoO_x_/Glassy carbon	-	-	-	-	-	-	[114]
	Pear	Fe_3_O_4_/Chitosan nanoparticles	145 μg/mg	67.1%	-	-	Retained 90% of initial activity after 4 weeks storage at 4 °C	After 8 cycles, 85% of the initial enzyme activity was retained	[104]
	Mushroom	Polypyrrole nanotubes/GA	-	-	-	-	Retained 88% of initial activity after 30 days storage at 4 °C	-	[100]
	Banana	Acrylamide/N, N′-methylenebisacrylamided	-	-	-	-	Retained 100% of initial activity after 20 days storage at 4 °C	After 5 cycles of measurement, the RSD was 3.2%	[92]
	Mushroom	Polyaniline/Glutaraldehyde	-	-	-	-	Retained 80% of initial activity after 20 weeks storage at 4 °C	After 25 cycles of measurement, the RSD was 2.8%	[115]
	Mushroom	Silica/Acrylamide/Diacrylamide	-	-	-	-	Retained 100% of initial activity after 20 days storage at 4 °C	-	[116]
Covalent binding	Mushroom	Chitosan/Organic rectorite/Glutaraldehyde	26.5 mg/g					After 10 cycles, 73.2% of the initial enzyme activity was retained	[22]
Adsorption/Cross-linking	Plant tissue	Polyaniline/Porous polyacrylonitrile/Nanostructured graphene	-	-	-	-	-	After 10 cycles of measurement, the RSD was 3.9%	[86]
Adsorption/Covalent binding	Potato	Propylamine functionalized silica nanoparticles	-	-	-	-	Retained 40% of initial activity after 80 days storage at 4 °C	After 10 cycles of measurement, the RSD was 4.1% to 5.2%	[24]

Relative standard deviation: RSD.

### 3.4. Nanomaterials for Plant PPO Immobilization

With the development of nanomaterials, immobilized enzymes have been widely studied. Nanomaterials with high surface area and stability achieve high enzyme loading and retention [59]. Compatible nanostructures further enhance enzymatic stability, catalytic activity, and biocompatibility [117]. The nanomaterials used to immobilize plant-based PPOs over the years can be broadly categorized into metal nanoparticles, carbon-based nanomaterials, and nanocomposites, similar to those used for laccase immobilization [13]. As a commonly used immobilization material, silica has been studied for PPO immobilization on mesoporous carriers with different pore sizes and volumes. Pore size and volume were found to have a significant effect on PPO immobilization, with materials having wider pores and larger volumes exhibiting faster and higher loading capacity. The interaction force between PPO and the material can be influenced by pH to some extent, and the enhancement of activity varies between different PPO and material bindings [21,25]. Furthermore, amine-modified mesoporous silica improved PPO catalytic activity [69]. Therefore, selecting suitable pore size and volume seems necessary for optimizing immobilized PPO. CTS acts as an ideal support material with good biocompatibility and high affinity [118,119]. When combined with compatible AuNPs, it forms a carrier for immobilized PPO that exhibits higher activity [9]. CTS modified by GA increased the solidification rate and recycling times of PPO, showing good results for removing phenol, 4-chlorophenol, and 2,4-dichlorophenol from aqueous solutions [22]. In addition to removing phenolic pollutants, CTS composites with immobilized PPO can be used for metal ion concentration detection based on the effect of metal ions on PPO activity [26]. GO is a common electrode material for sensing and detection. Due to its good conductivity and stability, it can be combined with metal nanoparticles and other materials to immobilize PPO and construct electrochemical sensors based on voltammetry, which can be applied to detect phenolic compounds, dopamine, and other phenolic substances. The constructed sensors show good sensitivity and detection range [11,23,90,108]. GO also has good biocompatibility and can interact with PPO in fruits and vegetables to remove or inhibit PPO, prolonging freshness [25,73]. Additionally, the methyl orange-derived matrix is a good material for detecting phenolic contaminants based on voltammetry due to its biocompatibility and high surface area, with immobilized PPO retaining 88% of its original activity for 30 days [100]. The aforementioned amine-functionalized silica material combined with carbon material to adsorb potato-extracted PPO can also be used for phenol detection based on voltammetry [24,111]. Besides the above materials, polyaniline, chalcogenides, polypyrrole, and gels have been studied for phenol detection based on electrochemistry [84,92,108,115]. Researchers have also constructed fiber optic sensors that demonstrate high sensitivity, wide detection range, fast response, and repeatability in detecting phenolic pollutants [79,116]. SA, a natural polymeric material, can be combined with metal nano-ion particles to enhance PPO catalytic activity and phenolic pollutant degradation [77,78]. In these PPO immobilization composite studies, cross-linking agents like GA play an important role in connecting PPO to the material [99,100]. The continuous research and advancement of materials have promoted the improvement of immobilized PPO properties, broadened PPO applications, and enhanced the potential for PPO industrialization. Metal–organic frameworks have recently received significant attention due to their advantages such as controllable pore size, high surface area, diverse architectures, and high stability. Researchers have applied them with immobilized laccase for removing phenolic pollutants and in biosensing [13,66,120]. However, there are fewer applications in immobilizing plant PPO. This will be a good research guide to make plant PPO play a better role in environmental treatment, bioassay, and theaflavin synthesis.

## 4. Multi-Field Applications of Plant PPO Immobilization

**Figure 2:**
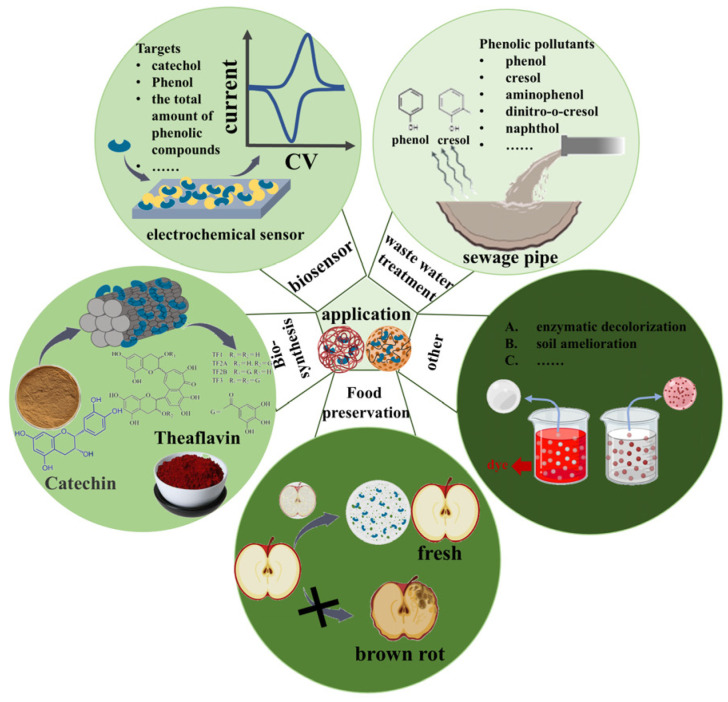
Application of plant PPO.

With the rapid development of immobilization technology, the enzymatic activity, stability, and reusability of PPO have been significantly improved. This makes immobilized PPO better suited for applications in wastewater treatment, environmental phenolic detection sensors, and the synthesis of active substances (Figure 2) [9,10].

### 4.1. Wastewater Treatment

Phenolic compounds and their derivatives, as common environmental pollutants, are hazardous and resistant to degradation, causing serious damage to natural flora and fauna. Being highly soluble in water, they are widely found in industrial wastewater [55,121]. Prolonged exposure of humans to excessive phenolic compounds can potentially induce cancer and lead to the development of chronic diseases such as kidney disorders and tumors [122,123]. Therefore, the removal of phenolic compounds from wastewater has become an urgent problem. PPO is one of the enzymes studied as a catalyst for the conversion of phenolic hydroxyl groups to o-quinones and their removal by precipitation or filtration to purify wastewater. In recent years, the combination with immobilization technology has become one of the most important tools for the removal of phenolic compounds [69].

AbPPO4 was immobilized on SBA-15 and amino-functionalized SBA-15-NH_2_ by adsorption to oxidize four phenolic compounds. Compared with free AbPPO4, the immobilized PPO showed a maximum increase of about 11-fold in the removal rate of phenolics, which was greatly accelerated. SBA-15-PPO retained 63% of the initial enzyme activity after 15 cycles of serial oxidation of phenolics, demonstrating good stability and catalytic activity for removing these compounds. Removal of phenols by PPO immobilized (IPPO-Au) on a composite of CTS-AuNPs/MMT can be improved by optimizing the conditions [69]. The team of Li et al. successively optimized the immobilization parameters using the Taguchi method and Taguchi–Grey relational analysis. After optimizing the parameters by the Taguchi method, the removal rates of phenol, 4-chlorophenol, and 2,4-dichlorophenol by IPPO-Au reached 89.2%, 95.2%, and 93.8%, respectively, at 480 min, when only 7.2% of free PPO activity remained [72]. The removal of phenol, 4-chlorophenol, and 2,4-dichlorophenol by IPPO-Au reached 86.2%, 95.2%, and 93.1%, respectively, at 240 min after optimization of the parameters by Taguchi–Grey relational analysis [9]. The optimized immobilization parameters of the second method achieved faster and more active removal of phenolics from the wastewater. Therefore, the choice of immobilization parameters significantly enhances wastewater treatment.

In another study, Zhong et al. (2021) optimized the immobilization parameters of PPO immobilized on CTS/OREC by adsorption and covalent immobilization using the Taguchi method [22]. They found that phenol, 4-chlorophenol, and 2,4-dichlorophenol removals reached 69.3%, 89.8%, and 93.8%, respectively, at 120 min, and that adsorption immobilization was better than covalent immobilization for phenolic compound removal. Phenol was removed from wastewater by immobilizing peroxidase and PPO extracted from tea leaves using alginate-coated magnetic nanoparticles. The phenol removal rate of the immobilized enzymes reached 98.1% at 145 min, and the enzymes retained about 50% of their initial activity after six cycles in the phenol removal process, indicating that alginate magnetic nanocatalysts are effective for phenol removal from wastewater [10]. Edalli et al. (2016) immobilized PPO extracted from mushrooms using silver alginate nanoparticles to remove the pollutant p-cresol [77]. The immobilized PPO degraded a 20 mM solution of p-cresol twice as much as the free PPO, and the degradation ability remained intact after 12 cycles of reuse. Potato PPO was ligand-immobilized on activated cellulose with amino groups, and an aqueous solution containing 100 mg/L of 4-chlorophenol and 4-bromophenol showed a removal rate of more than 90%, which could be sustained for as long as 8 h [94]. Various studies have shown that the physical and chemical properties of the carrier materials used for immobilization differ, and different material carriers, immobilization methods, and immobilization parameters result in some differences in the activity of removing different phenolic compounds from wastewater.

### 4.2. Biosensors

Phenolic compounds and their derivatives, as substances that are difficult to degrade, are widely present in the natural environment, ecosystems, and household waste, and have irreversible harmful effects on living organisms [55,108]. Although various methods and instruments (e.g., chromatography, spectrometry, and spectrophotometry) have been developed for determining the number of phenolic compounds and are widely used in environmental and wastewater monitoring, they are expensive and time-consuming [124,125,126]. Consequently, research focus has shifted toward developing economical, rapid, and precise biosensors for phenolic bioassays [79,127,128]. Bio-detection sensors are mainly composed of two parts: a biochemical recognition system and a sensor [13]. They can contact the detected samples, transfer biochemical information into electrical or other output signals, and the sensor senses the signal size change to analyze the amount of phenolics. PPO can enzymatically react with phenolics, resulting in changes in the system’s microenvironment, such as electron transfer and substrate change. This makes it an important medium for biosensing applications. In recent years, combining immobilization to improve the activity, stability, and reusability of PPO to provide a stable biosensor platform for detecting phenolics has been widely studied. Based on the fast electron transport, excellent electrical conductivity, and good mechanical strength of nanostructured graphene, Zheng et al. (2019) constructed a biosensor platform with enhanced stability based on current response by embedding PPO into nanostructured graphene hybridized components [108]. The biosensor achieved a sensitivity of 6.46 μAμM^−1^ cm^−2^ and a detection limit of 2.65 × 10^−7^ M, which was much higher than that of biosensors with similar carriers [129,130,131]. The difference in the detection limit of this biosensor was small compared with that of UPLC. Kıranşan et al. (2020) also constructed a phenolics detector based on the immobilization of potato-derived PPO at polyglycine/rGO paper electrodes using graphene as a base for detecting catechols, with a sensitivity of 51.2 uA µMcm^−2^ over a concentration range of 0.1–800 µM and a limit of detection of 7 × 10^−5^ M, a performance slightly lower than the sensor studied by Zheng et al. (2019) [11,108]. Mishra et al. (2020) prepared a biosensor with a fiber grating of polyacrylamide gel-embedded PPO for detecting phenolic pollutants in the environment [79]. It was designed to respond to the concentration of phenolics by changing the magnitude of the refractive index of the gel by the product, which induces a resonance shift, with a sensitivity of 0.0088 nm/μM. Their team investigated the detection of environmental phenolics by polyacrylamide gel-embedded PPO based on surface plasmon resonance shifts back in 2013, with a sensitivity of about 0.03 nm/μM, which is lower than that of biosensors with fiber gratings [116]. A novel polypyrrole nanotube cross-linked with PPO with methyl orange reference was used as a phenolic biosensor with a sensitivity of 2.981 µA M^−1^ cm^−2^ and a detection limit of an impressive 1.22 × 10^−9^ M, and still retained 88% of the original activity after 30 days of operation. It is currently a more excellent detector for phenolic compounds [100]. Biosensors with immobilized PPO can also be used to detect phenolic dopamine with detection limits of 8 × 10^−9^ M [23].

PPO, as the main enzyme produced by enzymatic browning, can irreversibly harm the organoleptic quality of foods such as fruits and vegetables. However, the emergence of inhibitors has been beneficial. By adding inhibitors, the enzymatic browning of foods such as fruits and vegetables can be reduced, preserving their nutritional value and sensory quality [113,132]. But excessive additions of inhibitors can be seriously harmful to human health and even life-threatening [133,134,135]. Therefore, it is necessary to monitor the number of inhibitors in foods to maintain compliance with food additive-related regulations. Excess or residual inhibitors can be reflected by inhibiting the activity of PPO-catalyzed phenolics, and constructing PPO-immobilized biosensors for rapid detection of inhibitor amounts has become a simple, rapid, and accurate method. do Nascimento Marreiro Teixeira et al. (2020) immobilized PPO on CTS-coated graphite electrodes by GA cross-linking and used voltammetry to measure the activity changes in this biosensor [113]. Their biosensor showed a sensitivity of 2.18 μA/μmol L^−1^ and a detection limit of 0.151 uM with good stability. A rapid autoradiographic method for enzymatic browning inhibitor detection on thin layer chromatography of immobilized PPO was achieved for detecting the number of natural inhibitors [80]. Since different metal ions affect PPO activity differently, a bio-detector based on CTS-gelatin biocomposites with immobilized banana PPO can also be used to detect metal content and still maintain 80% of the original activity after 30 days [26]. It can be seen that biosensors based on immobilized PPO have good application prospects for detecting both phenolic compounds and food inhibitors.

### 4.3. Food Preservation

As described in Section 3.1.1, enzymatic browning induced by PPO can lead to negative impacts on the flavor and nutritional value of foods such as fruits and vegetables, and even cause spoilage [27]. Although inhibitors such as sulfites, benzoic acid, and flavonoids can inhibit PPO activity, excessive residues can be harmful to humans [136,137]. Researchers are also attempting to inhibit PPO activity through refrigeration or non-thermal treatment techniques, which have shown promising results, but with economic costs and changes in the organoleptic properties of the food [138]. Although immobilization can improve PPO-related properties for the removal of phenolics from the environment, highly stable and high surface area immobilized materials interacting with PPO can also prevent reactions with phenolic compounds and have good application in inhibiting PPO activity. Corell Escuin et al. (2017) utilized the strong adsorption of modified mesoporous silica materials to PPO to form an isolation with phenolic compounds [25]. The SB15-type material can inhibit 50% of PPO activity in 15 min, which is promising for application. GO and rGO materials have high surface area and biocompatibility, which can be removed from fruits and vegetables by irreversible bonding with adsorbed PPO. PPO adsorbed by GO and rGO materials can be stably stored for one month in vitro [74]. Although immobilization can effectively inhibit PPO activity and protect food from browning, it requires a high degree of safety and performance of the carrier material, and its development remains a direction that needs long-term exploration.

### 4.4. Theaflavin Synthesis

Theaflavin, a component of black tea, is synthesized from catechins through PPO-catalyzed synthesis. It has four main components: theaflavin (TF1), theaflavin-3-gallate (TF2a), theaflavin-3′-gallate (TF2b), and theaflavin-3,3′-digallate (TF3) [5,139]. Beyond their sensory contributions, theaflavins exhibit significant bioactive properties including antioxidant, anti-inflammatory, antidepressant, and cardioprotective effects [140,141,142]. Based on these health benefits, functional products containing theaflavins have been gradually developed, such as beverages and other health products [143,144]. However, the proportion of theaflavins in black tea is relatively low, and the amount of theaflavins extracted directly from black tea does not meet consumer demand [145,146]. Moreover, chemically synthesized theaflavins have problems such as low purity, poor selectivity, and environmental pollution [5,147]. Catechins in PPO-fermented black tea can increase the yield of synthesized theaflavins in a green and efficient manner [21,145]. Therefore, research on the synthesis of theaflavins by PPO has rapidly developed, and PPO from sources such as tea, pear, and potato has been used to synthesize theaflavins. PPO mainly oxidizes catechol-type catechins to form quinones, which then undergo a coupling reaction followed by non-enzymatic condensation to form theaflavins [5,146]. However, free PPO can suffer from poor enzyme activity, low stability, and non-reusability [69,72]. Enzyme immobilization has been applied to enhance the properties associated with PPO to improve the efficiency and yield of fermented theaflavins. Lei et al. (2017) covalently immobilized PPO purified from pears onto Fe3O4/GA nanoparticles to efficiently synthesize TF3 using epigallocatechin-3-O-gallate and epigallocatechin gallate as substrates [104]. The maximum yield of TF3 reached 42.23%, and the immobilized PPO retained 85% of its original activity after eight consecutive cycles of use, with a storage time of up to 30 days. The immobilization of PPO with mesoporous silica is not only effective in removing phenolics and inhibiting enzymatic browning but also plays an important role in the synthesis of theaflavins. Zeng et al. (2019) immobilized nine PPOs purified from *Agaricus bisporus*, sweet potato, and grape on mesoporous silica to produce theaflavins with tea polyphenols as substrates [21]. Eight immobilized PPOs could produce theaflavins with the highest activity, reaching two times that of the free enzyme. There was also a study based on polyethylene glycol-immobilized PPO to synthesize theaflavin with catechins, and although the yield of TF3 could reach 102 μg/mL, the PPO could only maintain 50% of the original activity after four cycles [103]. Although plant-derived PPO has been widely tapped, the fermentation of immobilized PPO for the production of theaflavins can be limited by factors such as the biocompatibility and stability of the material carriers, as well as the safety of the synthesized theaflavins, making the industrial synthesis of theaflavins from immobilized PPO with high efficiency and high recycling a long-term challenge.

### 4.5. Other Applications

In addition to the four main applications mentioned above, immobilized PPO has also been used to catalyze the decolorization and decontamination of organic dyes in wastewater. Although much research in this area has focused on immobilizing laccase for decolorization [148], immobilization of purified plant PPOs has also been explored. Arabaci et al. (2014) immobilized PPO purified from papaya leaves on calcium alginate beads for the removal of synthetic dyes from industrial wastewater [95]. Immobilization improved the thermal stability of PPO compared to the free enzyme. Decolorization of eight different textile dyes was carried out, and the decolorization rate of immobilized PPO increased for all dyes at pH 4, with the highest rate increasing by 57.43% compared to free PPO, demonstrating significant decolorization efficiency. In removing polycyclic aromatic hydrocarbons (PAHs) from soil, the activity of PPO is increased by adsorption and immobilization on activated carbon, which appears to achieve soil remediation [149].

## 5. Future Prospects

In summary, immobilization of plant PPO on diverse composite materials has significantly enhanced its thermal, pH, and solvent stability while improving reusability and expanding application scope. However, immobilization also suffers from issues such as negative effects on PPO activity and detachment of immobilized PPO after cycling, which seriously impact further improvements in immobilization efficiency. The impact on PPO activity is mainly due to the effect of immobilization on enzyme conformation and flexibility. Therefore, future advances necessitate developing bioactive composites that preserve enzyme flexibility for industrial implementation. With the emergence of various novel metal- and carbon-based materials, enzyme immobilization is expected to reach new levels, achieving good compatibility between materials and immobilized enzymes. Currently, immobilized PPO has also been used in combination with nanomaterials in fields such as environmental remediation, sensing, and detection. Due to the green nature of immobilized PPO, it has become a new-generation technology to address these areas by replacing polluting chemical methods and ineffective physical methods. Unfortunately, it has failed to be industrialized and remains largely at the laboratory scale for small-scale exploration, and the further utilization of abundantly sourced plant PPOs has not met expectations. Therefore, we need to leverage the advantages of immobilization to expand the scale and batch application in the environment, food, and testing fields to fully harness the potential of PPO.

## 6. Conclusions

In conclusion, this review summarizes the research progress and applications of combining plant PPO with immobilization techniques in plants. Immobilized plant PPO can improve its stability, recyclability, and reusability, and enhance catalytic efficiency and economy. Through physical adsorption, encapsulation, chemical cross-linking, and covalent binding, plant PPO has been successfully immobilized on a variety of carriers, demonstrating a wide range of potential applications in environmental remediation, biosensor development, food preservation, and theaflavin synthesis. Although immobilized plant PPO technology has made remarkable progress, it still faces challenges such as the identification of carrier materials, optimization of immobilization parameters, and expansion of application scale. Future research needs to further explore novel immobilization materials, improve immobilization techniques, and expand the application of immobilized plant PPO in more fields to achieve maximum utilization of it in industrial applications.

## Data Availability

No new data were created or analysed in this study. Data sharing is not applicable to this article.
